# Malignant Disease of the Vocal Cords

**Published:** 1898-10-29

**Authors:** 


### MALIGNANT DISEASE OF THE VOCAL CORDS.

Dr. Herbert Tilley* relates two cases of epithelioma of the vocal cord, in both of which the growth was removed by thyrochondrotomy, and in one of them no recurrence had taken place after two years. He describes the steps of the operation and shows that the dreaded complication of septic pneumonia, which was the cause of its great mortality in former times can now be almost certainly avoided, and that the risk of the operation is thus so diminished that it may properly be recommended in the earliest stage in which the disease can be recognised instead of being kept for a last resource. Indeed, the difficulty is to discover the disease early enough, for at this practically curable stage the symptoms may be very slight, nothing more in fact than mere hoarseness, of which but little notice may be taken either by patient or medical man. Such cases thus tend too often to drift on until it is too late to undertake any radical treatment with even a fair prospect of success. In the first case related a man aged 65 had had hoarseness for from 12 to 14 months, but he had no pain or difficulty in swallowing and no perceptible loss of flesh, and he expressed himself as "ashamed to consult anyone for such a small matter." Yet, on examination with the laryngoscope the right vocal cord was found thickened, ulcerated, and congested, the upper edge of the ulcer being thickened. The cord also was immobile on phonation. At the operation the whole of the vocal cord, together with the right vocal process and the right arytenoid cartilage had to be removed. The second case was a man, aged 49, and he also complained only of "hoarseness" of two months' duration. On laryngoscopic examination, however, a whitish grey nodular thickening was discovered, occupying the anterior fourth of the left vocal cord. At its junction with the cord posteriorly the latter was distinctly congested, in marked contrast to the colour of the corresponding part of the right vocal cord. After the operation in this case the man had an excellent voice. The lesson of importance which such cases teach is that a middle-aged man, even in apparently perfect health, but suffering from "hoarseness" of more than temporary duration, or even marked alteration of the voice, should not be allowed to rest until his larynx has been carefully examined by some one skilled in the use of the laryngoscope. "It is by the general practitioner that this point should be borne in mind, for as a rule it is he who is first consulted in the matter, and if he prescribes gargles, pastiles, &c., waiting to make his diagnosis from pain 'shooting from the throat to the ear,' pain or difficulty in swallowing, &c.—symptoms which only, as a rule, occur late in the disease—he will probably rob his patient of his only chance of cure."

* Brit. Med. Jour., Oct. 22.

